# Menthol Flavor in E-Cigarette Vapor Modulates Social Behavior Correlated With Central and Peripheral Changes of Immunometabolic Signalings

**DOI:** 10.3389/fnmol.2022.800406

**Published:** 2022-03-10

**Authors:** Zhibin Xu, Ye Tian, A.-Xiang Li, Jiahang Tang, Xiao-Yuan Jing, Chunshan Deng, Zhizhun Mo, Jiaxuan Wang, Juan Lai, Xuemei Liu, Xuantong Guo, Tao Li, Shupeng Li, Liping Wang, Zhonghua Lu, Zuxin Chen, Xin-an Liu

**Affiliations:** ^1^Guangdong Provincial Key Laboratory of Brain Connectome and Behavior, CAS Key Laboratory of Brain Connectome and Manipulation, Brain Cognition and Brain Disease Institute (BCBDI), Shenzhen Institute of Advanced Technology, Chinese Academy of Sciences, Shenzhen, China; ^2^Shenzhen-Hong Kong Institute of Brain Science-Shenzhen Fundamental Research Institutions, Shenzhen, China; ^3^University of Chinese Academy of Sciences, Beijing, China; ^4^Department of Forensic Medicine, School of Medicine, Xi’an Jiaotong University, Xi’an, China; ^5^State Key Laboratory of Oncogenomics, School of Chemical Biology and Biotechnology, Peking University Shenzhen Graduate School, Shenzhen, China; ^6^Department of Psychiatry, University of Toronto, Toronto, ON, Canada; ^7^Key Laboratory of Modern Toxicology of Shenzhen, Shenzhen Center for Disease Control and Prevention, Shenzhen, China; ^8^Shenzhen Key Laboratory of Drug Addiction, Shenzhen Neher Neural Plasticity Laboratory, The Brain Cognition and Brain Disease Institute, Shenzhen Institute of Advanced Technology, Chinese Academy of Sciences, Shenzhen, China

**Keywords:** e-cigarette, nicotine, menthol, social activity, electronic nicotine delivery systems (ENDS)

## Abstract

The use of electronic cigarette (e-cigarette) has been increasing dramatically worldwide. More than 8,000 flavors of e-cigarettes are currently marketed and menthol is one of the most popular flavor additives in the electronic nicotine delivery systems (ENDS). There is a controversy over the roles of e-cigarettes in social behavior, and little is known about the potential impacts of flavorings in the ENDS. In our study, we aimed to investigate the effects of menthol flavor in ENDS on the social behavior of long-term vapor-exposed mice with a daily intake limit, and the underlying immunometabolic changes in the central and peripheral systems. We found that the addition of menthol flavor in nicotine vapor enhanced the social activity compared with the nicotine alone. The dramatically reduced activation of cellular energy measured by adenosine 5′ monophosphate-activated protein kinase (AMPK) signaling in the hippocampus were observed after the chronic exposure of menthol-flavored ENDS. Multiple sera cytokines including C5, TIMP-1, and CXCL13 were decreased accordingly as per their peripheral immunometabolic responses to menthol flavor in the nicotine vapor. The serum level of C5 was positively correlated with the alteration activity of the AMPK-ERK signaling in the hippocampus. Our current findings provide evidence for the enhancement of menthol flavor in ENDS on social functioning, which is correlated with the central and peripheral immunometabolic disruptions; this raises the vigilance of the cautious addition of various flavorings in e-cigarettes and the urgency of further investigations on the complex interplay and health effects of flavoring additives with nicotine in e-cigarettes.

## Introduction

The use of electronic nicotine delivery systems (ENDS), also known as electronic cigarettes (e-cigarettes) has been dramatically increasing in recent years, and it has become a serious public health issue. Despite the lack of either health data or the demonstrated efficacy in promoting smoking cessation, the e-cigarette is often advertised as a safer alternative or cessation aid to conventional tobacco cigarette smoke, mainly due to its much lower levels of toxic/carcinogenic chemicals (Arnold, 2014; Benowitz, 2014; Ramamurthi et al., 2016). Although the popularity of e-cigarette use continues to increase, research evidence based on scientific knowledge is lacking and the main focused aspect of e-cigarettes include their beneficial roles in tobacco smoking cessation or reduction, their health risks, and their environmental consequences (Rom et al., 2015; Hartmann-Boyce et al., 2020).

The major composition of e-cigarettes usually consists of propylene glycol and vegetable glycerol (PG/VG) as odorless liquid vehicles to generate vapor, nicotine which is the main addictive substance, and a wide variety of flavorings (Allen et al., 2016; Smith et al., 2020). As the number of users grows exponentially worldwide, liquids of e-cigarettes are available in a dramatically large combination of flavor additives, with more than 8,000 flavorings (Zhu et al., 2014; Hsu et al., 2018). A recent increase in the prevalence of e-cigarettes among young adults and adolescents may largely be due to their widely available flavors which appeal to the youth (Ambrose et al., 2015; Villanti et al., 2017; Cullen et al., 2019a,b). Epidemiological survey data have shown that the most common flavor categories include fruit, menthol, and tobacco. Menthol flavor was shown to be one of the most popular flavors among young users (Leventhal et al., 2019) and the extent of satisfaction with vaping varies among unique flavor users (Gravely et al., 2020; Rose et al., 2020). However, the incorporated effects of flavorings when added in the nicotine-containing vapor and the underlying mechanisms are largely unknown.

Many previous studies have confirmed the association between cigarette smoking and neurodegeneration (Deochand et al., 2016; Yu et al., 2016; Liu et al., 2020), cognition and memory (Ge et al., 2019; Wei et al., 2020; Martin Rios et al., 2021), and mental disorders, such as attentional deficits (Joo et al., 2017; Lawrence et al., 2021) and schizophrenia (Donde et al., 2020; King et al., 2020), considering the wide distribution of nicotinic acetylcholine receptors (nAChRs) throughout the brain (Levin et al., 2015). Based on the fact that nicotine is one of the main addictive components of an e-cigarette, there is increasing recognition that e-cigarettes impact brain functions, for instance, e-cigarettes impaired the integrity of the blood-brain barrier (BBB) and exacerbated the cognitive dysfunction (Chen et al., 2021), mental disorders (Pham et al., 2020), vascular inflammation (Kaisar et al., 2017), metabolic imbalance (Debarba et al., 2020), and neurotoxicity (Ruszkiewicz et al., 2020) in the brain of human and animal models, while the effects of ENDS with specific flavor on the behaviors need further disclosure. Furthermore, it is the utmost emergency to understand the molecular architectures sculptured in the brain and the peripheral system that synergistically respond to the ingredients of e-cigarettes.

The aim of the current study is to provide a comprehensive behavioral analysis of ENDS with menthol flavor in male mice (Leventhal et al., 2021). We sought to characterize the impacts of the immunometabolic signals on the key brain regions that may account for the behavioral changes. The proteomic cytokine array of the serum was also investigated to detect the circulating immunological signals that were influenced by menthol flavor in ENDS. The correlation analyses among the behavioral parameters and the central and peripheral immunometabolic indices were conducted to reveal the systemic responses mediated by menthol flavor in ENDS.

## Materials and Methods

### Animals

Male C57BL/6J mice (Hunan SJA Laboratory Animal Co., Ltd., Hunan, China) aged 8 weeks old, were maintained in standard housing conditions on a 12/12 h day/night cycle (lights on at 7 a.m. and off at 7 p.m.) with *ad libitum* access to food and water. All behavioral tests were conducted at a fixed time period during the light cycle. All mice were handled for 15–20 min per day for 3 days before behavioral assays to reduce the stress introduced by contact with an experimenter. All animal experiments and procedures were carried out in accordance with the protocols approved by the Animal Care and Use Ethics Committee of the Shenzhen Institutes of Advanced Technology, Chinese Academy of Sciences.

### E-Cigarette Exposure System

**FIGURE 1:**
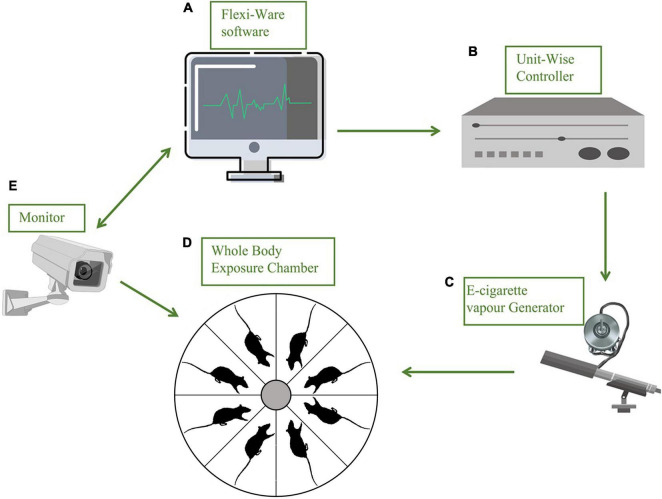
Schematic diagram of a precise substance vapor device. Each part of the device is represented by a stick figure. **(A)** Flexiware software: the exposure temperature, patterns of smoking and humidity, and the exposure time and duration can be modified practically by the computerized exposure system; **(B)** a Uint-Wise controller connected with **(C)** e-cigarette vapor generator; **(D)** whole body exposure chamber with relatively spacious space, eight mice for every single run, and the action state of animals in the chamber was recorded in real-time by a camera **(E)** connected with **(A)**; a gas purifier before it is discharged.

**FIGURE 2:**
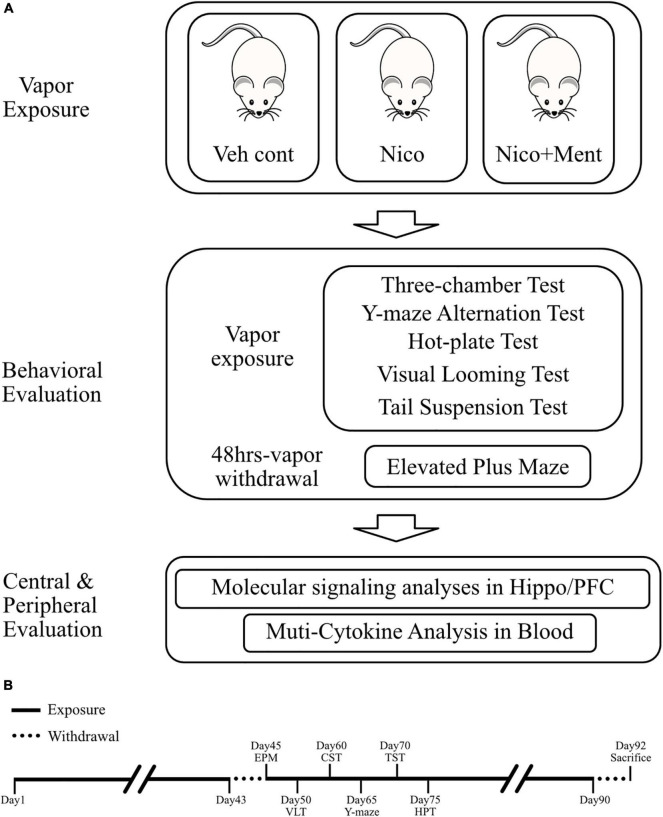
Experimental design of this study. **(A)** Diagram of experimental procedures. The duration of vapor exposure is 30 min per day and 7 days a week. Veh cont, Vehicle control of propylene glycol/vegetable glycerin; Nico, Nicotine; Nico + ment, nicotine with menthol flavor. **(B)** Schematic workflow of this study. Veh cont, Vehicle control of 50% propylene glycol (PG) + 50% of vegetable glycerin (VG); Nico: 4% of Nicotine + 46% of PG + 50% of VG; Nico + ment: 10% of menthol flavoring agent + 4% of Nicotine + 35% of PG + 51% of VG.

The inExpose e-cigarette device (SCIREQ Scientific Respiratory Equipment Inc.) was used in our experiments for vapor exposures of accurate amounts of nicotine between groups, as shown in Figure 1. During the experiments, 8 mice were treated in one single run, in a closed whole body exposure chamber with relatively spacious space. The action state of animals in the chamber was recorded in real-time by a camera connected to a computer. A supporting software (IX-2PD-4DIO-ECIG inExpose) was applied for controlling the e-cigarette vapor generation and postprocessing. For details of the running program, the exposure duration was set as 30 min per run, one time per day. The gas flow rate was set as 2 L per min (Supplementary Table 1). The purified exhaust gas was expelled after the exposure. The e-cigarette vapor exposures were carried out for 7 days per week, for 43 days, and the following behavioral tests were performed with a continuous daily vapor exposure except that the elevated plus maze was assessed when the ENDS had been withdrawn for 48 h (Figure 2). The mice were randomly assigned to one of the three treatment groups and exposed daily as follows: (1) Veh cont: Vehicle control of 50% propylene glycol (PG) + 50% vegetable glycerin (VG); (2) Nico: 4% Nicotine + 46% PG + 50% VG; (3) Nico + ment: nicotine with menthol flavoring: 10% of menthol flavoring agent + 4% of Nicotine + 35% of PG + 51% of VG. All behavioral tests were conducted approximately around 15–16 h following the e-cigarette exposure to avoid its acute effects, except the Elevated plus maze test performed at the 48 h withdrawal period.

### Behavioral Assessment

#### Three-Chamber Sociability and Social Novelty Test

The three-chamber test (TCT) is widely used to observe the sociability and social novelty of rodents. An opaque white box (42 cm length × 60 cm width × 25 cm height) was made of acrylic and each chamber measured 42 cm length × 20 cm width × 25 cm height. Before the test, the mice were placed in the corner of the center chamber to habituate to the 3 chambers and two empty cups for 10 min. In the TCT, a subject mouse is allowed to explore two opposing chambers containing another mouse (social stimulus) or empty cage in the sociability test; and to explore two opposing chambers containing the familiar mouse or the novel mouse in the test of preference for social novelty. In the first session (sociability test), the test mice were placed in the corner of the center chamber, a new male mice of the same age were placed into the cup in the left chamber, while no mice were placed in the right chamber. In the second session (social memory test), the mice in the left chamber remained unchanged, and another set of new male mice of the same age was placed into the cup in the right chamber. Each session was monitored for 10 min. Time around and the number of interactions with each wire cage, which either housed the mice or not, and the time spent in each chamber zone was recorded. The contact zone was considered to be at a 2-cm distance from each cup. The 3-chamber box and the cups were cleaned with 70% of ethanol between the sessions.

For sociability sessions,


Preferenceindex=Timearoundstranger 1Timearoundstranger 1+Timearoundemptycage

Preferenceofchamber=Timeinstranger 1chamberTimeinstranger 1chamber+Timeinemptycagechamber.

For social novelty sessions,


Preferenceindex=Timearoundstranger 2Timearoundstranger 1+Timearoundstranger 2

Preferenceofchamber=Timeinstranger 2chamberTimeinstranger 1chamber+Timeinstranger 2chamber.

#### Elevated Plus Maze

To measure the anxiety levels in e-cigarette withdrawn mice, the elevated plus maze (EPM) was assessed by using a plastic elevated plus maze constructed from two white open arms (25 cm length × 5 cm width) and two white enclosed arms (25 cm length × 5 cm width × 15 cm height) extending from a central platform (5 cm length × 5 cm width) at 90° which form a plus shape. The maze was placed 65 cm above the floor. A camera was set directly above the EPM apparatus for video recording. The mice were individually placed at the center, with their heads facing the open arms. The number of entries and the amount of time spent in the same type of arms were recorded during the 5-min sessions.

#### Tail Suspension Test

The tail suspension test (TST simulates the behavioral despair states similar to depression, and this behavioral test was performed as described before (Can et al., 2012; Liu et al., 2021). After 1 h of habituation in the experimental environment, each mouse was suspended on a metal bar 50 cm above the floor of the suspension box with an adhesive tape placed approximately 1 cm from the tip of the tail for 6 min. At the beginning of the test, the animals exhibited escape behaviors, which after a period of struggle, became more subtle. These subtle movements were considered as the immobility time. Immobility was defined as the absence of any limb or body movements, except those caused by respiration. The activities of the mice were recorded by a camera, and the immobility time during the 6-min testing period was calculated. During the test, the mice were recorded separately to prevent animals from observing or interacting with each other. After each animal had completed the test, the suspension box was thoroughly cleaned to eliminate olfactory effects.

#### Visual Looming Test

The visual looming test (VLT) was performed in a closed Plexiglas box (40 cm length × 40 cm width × 30 cm height) with a sheltered nest in the corner. For upper field looming stimulus (LS), an LCD monitor was placed on the ceiling to present multiple LS, which was a black disc expanding from a visual angle of 2° to 20° in 0.3 s, expanding the speed of 60° per second. The expanding disc stimulus was repeated 15 times in quick succession (totally 4.5 s). This together with a 0.066 s pause between each repeat forms the total upper visual field LS that lasts 5.5 s. Behavior was recorded using an HD digital camera (Sony, Shanghai, China). The latency between the placement and the first overt behavioral signs, such as escape behavior and time staying in the nest were recorded. Animals were handled and habituated for 10–15 min in the looming box 1 day before testing. During the looming test session, the mice were first allowed to freely explore the looming box for 5 min. No observable adaptation was observed in all our experiments.

#### Hot-Plate Test

To measure the basal responsiveness to nociceptive stimulation, the mice were placed on a hot-plate set at 55 ± 1°C. The antinociceptive response was the latency from the placement of the mouse on the heated surface until the first overt behavioral sign of nociception, such as licking a hind paw, vocalization, or jumping off the plate. The time between the placement and the first overt behavioral sign was recorded as a pain threshold in this test and the mouse was immediately removed from the hot plate immediately after responding or after a maximum of 30 s (cut-off), to prevent tissue damage.

#### Y Maze Spontaneous Alternation Test

The Y maze test was conducted to detect spatial memory and spontaneous alternation performance. The Y maze used in this study is composed of three arms (42 cm length × 4 cm width × 25 cm height) projecting from a central triangular area. The mice were placed in the central area and were allowed to explore freely for 8 min. The observer recorded an arm entry when the hind paws were completely within the arm. Spontaneous alternation was defined as successive entries into the three different arms (without returning to any arm). The percentage alternation was calculated as the ratio of actual to possible alternations (the total number of arm entries - 2) × 100. The arms were cleaned with 70% ethanol between sessions.

### Tissue Processing and Western Blot

The mice were sacrificed immediately after behavioral experiments. They were anesthetized with isoflurane (0.3 ml per 25 g mouse) and euthanized by exsanguination. The brain regions of the frontal cortex and the hippocampus were dissected out on the ice and stored at -80°C for later use. The samples were homogenized in a Radioimmunoprecipitation (RIPA) lysis buffer with 1 time protease inhibitor cocktail and 1 time phenylmethylsulfonyl fluoride (PMSF). Homogenates were incubated on ice for 30 min and centrifuged at 12,000 × rpm for 10 min at 4°C. The concentration of total protein in each sample was measured using a bicinchoninic acid (BCA) kit. Then, the sample was mixed with 6 times loading buffer and boiled at 100°C for 10 min. The denatured samples containing 20 μg of total protein were separated by sodium dodecyl sulfate–polyacrylamide gel electrophoresis (SDS-PAGE), and then transferred to the nitrocellulose membrane. The membrane was blocked with 5% non-fat milk in Tris-buffered saline (TBST; 0.1% Tween 20) at room temperature for 1 h, then incubated in primary antibodies overnight at 4°C. The next day, the membrane was incubated with HRP-conjugated secondary antibodies for 1 h at room temperature. For detection, the ECL super signal chemiluminescence kit was used according to the manufacturer’s protocol. The gray intensity analysis of the bands was performed using Image J software (NIH, United States).

### Blood Collection and Proteome Profiler Mouse Cytokine Array

Orbital sinus blood samples were collected before sacrifice from the chronic ENDS-exposed mice. After collection, the blood samples were kept on ice and then centrifuged (3,000 rotations per minute for 10 min at 4°C), and the serum was separated. The serum samples were used fresh or kept at -80°C until further processing. The Proteome Profiler Mouse Cytokine Array Kit (Panel A; R&D Systems) was used to profile cytokines in 50 μL of serum samples according to the manufacturer’s protocol. The visualization of the array membranes was achieved using an enhanced chemiluminescence detection and exposure to X-ray film (Kodak, United States). Densitometry analysis was carried out using Quantity One.

### Urine Collection

To confirm the exposure of nicotine e-cigarettes in the appropriate treatment group, urinary cotinine levels in all the groups were measured at random days after aerosol exposure by using ultraperformance liquid chromatography coupled with tandem mass spectrometry (UPLC-MS/MS) method. Urine was collected during aerosol exposure from the plastic film located at the bottom of the exposure chamber using a pipette and transferred to a microcentrifuge tube. Separate films were replaced between groups in the exposure trials.

### Ultraperformance Liquid Chromatography Coupled With Tandem Mass Spectrometry Analysis

The UPLC–MS-MS analysis was performed on a Waters Acquity ultra-performance liquid chromatography (UPLC) system interfaced with a Waters Xevo TQ MS. Chromatographic separation of cotinine was achieved with an HSS T3 column (2.1 × 100 mm; 1.8 μm particle size). The temperature of the column was maintained at 40°C. A portion of 2.0 μL of the extracted sample was injected onto the column and the gradient elution was performed with 0.1% (v/v) formic acid in deionized water (mobile phase A) and (methanol mobile phase B) at a flow rate of 0.2 mL/min. The MS detection of cotinine was conducted by electrospray ionization (ESI) in the positive ion mode, using the multiple reaction monitoring (MRM) for analyte identification. The following ESI conditions were applied: capillary voltage of 1.5 kV; source temperature of 150°C; desolvation temperature of 400°C; and desolvation gas flow (nitrogen) of 800 L/h; The analysis time was approximately 5 min per sample. About 100 μL of the test urine sample was first diluted by 900 μL of ultrapure water in the centrifuge tube, and then eddied for 1 min and centrifuged at 10,000 rpm for 10 min. The supernatant was taken for testing.

### Statistical Analyses

Experiment data are expressed as the mean ± SEM of the number of tests stated. Statistical comparisons were made using one-way ANOVA followed by Tukey’s HSD test, as indicated in the figure legends. All the statistical tests were performed using the Prism 8.0 software (GraphPad Software Inc., San Diego, CA, United States). Spearman’s correlation analysis was used to conduct the correlations among the behavioral parameters and the AMPK activation in the hippocampus, as well as the altered cytokine levels in the sera. A *p*-value of less than 0.05 was considered statistically significant.

## Results

### Compensatory Enhanced Sociability in Mice Exposed to Electronic Nicotine Delivery Systems With Menthol Flavor

The e-cigarette products in the market usually offer a very wide variety of flavoring agents mixed with nicotine, which is one of the biggest health concerns of e-cigarettes (Rambaran et al., 2019; Malik et al., 2021). Here, we aim to evaluate whether the social functioning is modified by ENDS with a flavoring compound. Menthol is one of the most prevalent and common flavors used in e-cigarettes; so, we compared the behavioral responses in vapor-exposed mice between the nicotine alone group and nicotine group mixed with menthol flavoring. To do this, adult male C57BL/6J mice were randomly assigned to three treatment groups (*n* = 8 per group) which were exposed to the following: (1) propylene glycol and vegetable glycerol as vehicle control (50:50, PG/VG, Veh cont); (2) PG/VG with 4% (vol/vol) nicotine (Nico); (3) Vapor of 4% nicotine with 10% of menthol flavorings (Nico + ment). Here, we selected the inhalation model with a short duration (30 min) per day and long-term vapor exposure (>40 days) period. After a daily vapor exposure of 30 min and 7 days a week for 43 days, the three-chamber sociability and social novelty tests were evaluated between the above groups (Figures 2A,B). Since we focused to investigate the merged effects of menthol flavor in the nicotine vapor, here, we have only used the PG/VG as vehicle control in the following sets of behavioral assessments. To verify the exposure constituents, the levels of urine cotinine (i.e., nicotine metabolite) were assessed for the mice in all three vapor exposure groups. Mean cotinine levels averaged from multiple mice per exposure run on the random days were higher in urine from the mice exposed to vapors of nicotine alone (319.3 ± 33.99 ng/mL) or nicotine with menthol flavor (452.8 ± 92.71 ng/mL) compared to the Veh control (50.97 ± 13.36 ng/mL) [*F*_(2, 6)_ = 12.65, *p* = 0.0070, Supplementary Figure 1]. The body weight was measured weekly and no significant differences were observed in weight gain among all the treatment groups under our vapor exposure condition (Supplementary Figure 2).

**FIGURE 3:**
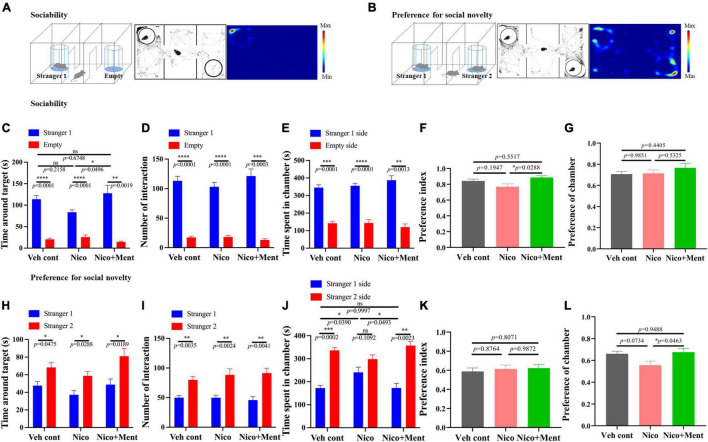
Social behavioral assessments in mice after long-term vapor exposure. Schematic diagram of two parts of the Three-Chamber Test (TCT); stage one **(A)** and stage two **(B)**. **(A,B)** Left: the schematic diagram of the sociability session and the social novelty session of the TCT. Center: the trajectory graphs represent the movements of the tested mouse during the sociability session and the social novelty session, respectively. Right: the heatmaps represent the trajectory of motions in mice. **(C–G)** Social behavioral assessments of tested mice in the sociability session. Time around target mouse [*F*_(2, 20)_ = 3.383, *p* = 0.0543] **(C)** and the number of interactions with stranger 1 [*F*_(2, 20)_ = 0.9427, *p* = 0.4062] or empty cage **(D)**. Time spent in the chambers of stranger 1 side or the empty side **(E)**. **(F)** Preference index of stranger 1 among groups. *F*_(2, 20)_ = 4.040, *p* = 0.0336. **(G)** Preference of stranger 1 chamber against the empty side among the exposure groups [*F*_(2, 20)_ = 0.9022, *p* = 0.4216]. **(H–L)** Social behavioral assessments of tested mice in the social novelty session. Time around target mouse **(H)** and the number of interactions [*F*_(2, 20)_ = 0.5234, *p* = 0.6004] **(I)** with stranger 2 mouse or stranger 1 mouse. **(J)** Time spent in the chambers of stranger 2 sides or the stranger 1 side. **(K)** Preference index of stranger 2 among the groups [*F*_(2, 20)_ = 0.2197, *p* = 0.8047]. **(L)** Preference of stranger 2 chamber against stranger 1 side among the exposure groups [*F*_(2, 20)_ = 4.081, *p* = 0.0326]. The calculations of Preference index and Preference of chamber are presented in Methods. Data are shown as mean ± SEM. *p < 0.05, ^**^p < 0.01, ^***^p < 0.001, and ^****^p < 0.0001 as determined by ordinary one-way ANOVA, within-group analyses using paired *t*-test, with the factors of test condition (sociability or social novelty), cages, and chamber sides (e.g., stranger 1 side or the opposite side). Veh cont, Vehicle control; Nico, Nicotine; Nico + ment, nicotine with menthol flavor.

Social interaction is a complex and highly conserved neuropsychiatric behavior that safeguards survival (Barak and Feng, 2016). Whether the additive of menthol flavor into e-cigarette would change the social interaction was unknown. We evaluated the social behaviors of mice *via* three-chamber social tests in this study. Sociability was investigated in the sociability session of the test (Figure 3A). Mice exposed to long-term ENDS with or without menthol flavor showed normal sociability as assessed by interaction time and time spent in the target chamber. Mice from the Nico group appeared normal while having slightly less contact and socialization with the stranger mouse 1 than in the empty arena compared with that in the Veh control group (No statistical significance, Figures 3C–E). Interestingly, the mice in the Nico + ment group prefer and spent more time (129.3 ± 21.01 s) to socialize with the stranger mouse 1 compared to the Nico group (83.67 ± 5.558 s, Figures 3C,F) while the time spent in the chamber of stranger mouse 1 and the preference of chamber was similar to the mice of all the groups [*F*_(2, 20)_ = 1.411, *p* = 0.2672, Figures 3E,G]. In the test session of preference for social novelty (Figure 3B), mice spent more time making contact and socializing with the newly introduced unfamiliar mouse (stranger 2) than stranger 1, as a normal manifestation of social memory and preference for social novelty [*F*_(2, 20)_ = 3.005, *p* = 0.0723, Figure 3H]. No significant difference were observed among the groups in their interacting time, the number of interaction, and the preference index with the novel mouse (Figures 3H,I,K), while mice exposed to nicotine alone spent less time in the chamber of the stranger 2 [*F*_(2, 20)_ = 4.542, *p* = 0.0236, Figure 3J], and mice of Nico + ment group also showed an increased preference for staying in the chamber of the newly introduced stranger 2 mice than with the familiar mouse, the stranger 1, compared to the mice in the group of nicotine vapor alone (Figures 3J,L). Our data suggested that long exposure to menthol-flavored ENDS may have compensatory enhancing effects on the sociability and preference for social novelty compared to the vapor exposure of nicotine alone.

### Effects of Long-Term Electronic Nicotine Delivery Systems Exposure With Menthol Flavor on Anxiety, Depression-Like Behaviors

**FIGURE 4:**
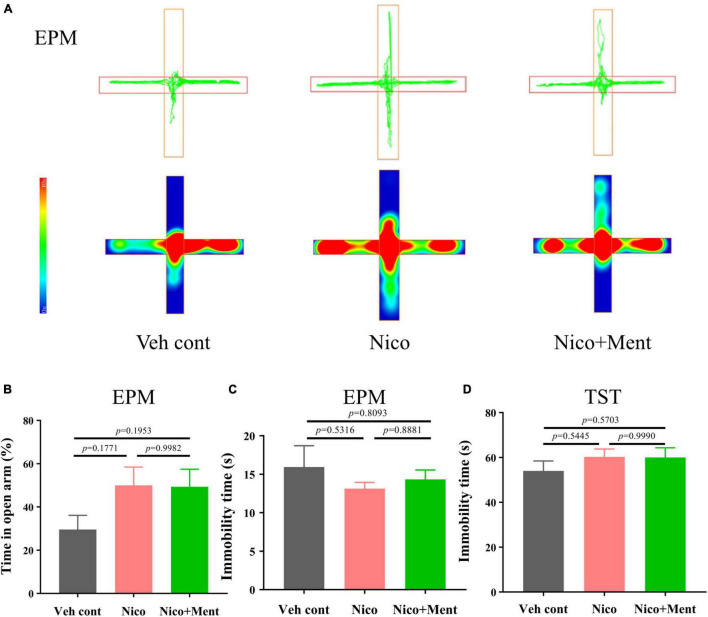
Anxiety, depressive-like behavioral assessments in mice exposed to long-term vapor exposure. **(A)** The trajectory and heatmap of mice in the EPM. **(B)** The percentage of time of staying in the open arm to the time of one trial ratio (%) [*F*_(2, 21)_ = 2.222, *p* = 0.1333]. **(C)** The immobility time of mice during the trial in EPM (s). **(D)** Immobility time of mice during Tail suspension test (s). The value of *p < 0.05 as determined by ordinary one-way ANOVA and multiple comparisons with every other group. Bars represent marginal means ± SEM. *N* = 8 per group. Veh cont, Vehicle control; Nico, Nicotine; Nico + ment: nicotine with menthol flavor. EPM, Elevated plus maze. TST, Tail suspension test.

The previous study has demonstrated the interplay between social behaviors and anxiety status (Lupien et al., 2009; Yamamuro et al., 2020). Since social stress is one of the major risk factors for the progression of anxiety disorders (Gross and Hen, 2004) (Feder et al., 2009), and shared neuronal circuits between them has been confirmed (Mottolese et al., 2014; Jing et al., 2021), we assessed the anxiety and the depression-like behaviors of the mice after long-term vapor exposure. We compared the 48 h-withdrawal responses in vapor-exposed mice with nicotine or nicotine plus menthol. By performing the behavioral tests of EPM (after 48 h-withdrawal) in mice, we observed that long-term vapor exposure of daily half-hour ENDS with or without menthol flavor did not cause significant withdrawal responses evaluated by anxiety-like behaviors in EPM at the 48-h-ENDS withdrawal period (Figures 4A,B). Specifically, we analyzed the immobility time for all groups during the EPM test [*F*_(2, 21)_ = 0.5963, *p* = 0.5599, Figure 4C; no significant differences were observed. Further, a tail hanging test was performed for the evaluation of depressive-like behaviors. No significant changes were observed on immobility time in the tail suspension test [*F*_(2, 21)_ = 0.7295, *p* = 0.4940, Figure 4D]. Our current data suggested that long-term e-cigarette usage with short daily nicotine exposure time did not induce anxiety or depression-like behaviors in mice, even after adding the menthol flavor in the e-liquid. These data suggested that the enhancement of menthol flavor in ENDS on social functioning is independent of the emotional status.

### No Innate Visual or Perceptual Behavioral Alterations in Mice After Long-Term Electronic Nicotine Delivery Systems Exposure With Menthol Flavor

**FIGURE 5:**
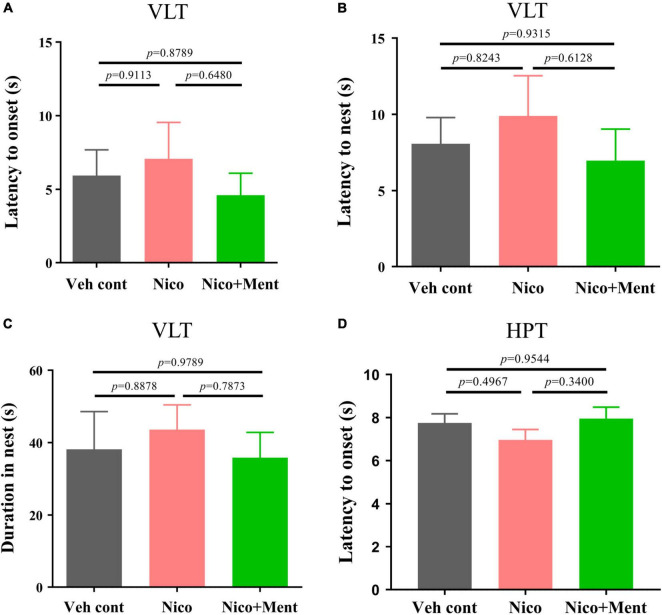
The innate visual or perceptual behavioral assessment in mice after long-term ENDS exposure. The behavioral tests were performed in order during ENDS exposure as described in Figure 2B. **(A)** The latency to the onset behavior of mice during the Visual looming test (VLT; s) [*F*_(2, 21)_ = 0.4033, *p* = 0.6732]. **(B)** The latency of flight to the nest behavior of mice during VLT (s) [*F*_(2, 21)_ = 0.4649, *p* = 0.6345]. **(C)** Duration of mice hiding in the nest (s) [*F*_(2, 21)_ = 0.2317, *p* = 0.7952]. **(D)** The latency to the onset behavior of mice during the Hot-plate test (s). The value of *p < 0.05 as determined by ordinary one-way ANOVA and multiple comparisons with every other group. Bars represent marginal means ± SEM. *N* = 8 per group. Veh cont, Vehicle control; Nico, Nicotine; Nico + ment, nicotine with menthol flavor.

We further evaluated whether the social functioning changes induced by the menthol flavor in ENDS are associated with any alternations on innate visual or perceptual behaviors (Ferrara et al., 2021; Sakurai, 2021; Wei et al., 2021). Behavioral paradigms, such as innate fear and heat pain response were used in our experiments. In the VLT, innate fear responses were quantified. Unexpected salient visual cues stimulate the animal’s defensive behaviors, such as shying away and hiding back in the nest (Zhou et al., 2019). We analyzed the onset latency of mice to such behaviors. There were no changes in the latency of flight to nest, the flight-to-nest latency, as well as the duration in the nest in the Nico group when compared to both Veh control and Nico + ment groups (Figures 5A–C). Chronic ENDS inhalation with daily limited-duration may not affect the innate fear responses in mice, and the same was also observed when menthol flavor was added. In the Hot-Plate Test (HPT), the pain response to a thermal stimulus was assessed by the onset of latency, and we found no differences among all groups [*F*_(2, 21)_ = 1.157, *p* = 0.3337, Figure 5D]. These data suggested that chronic nicotine vapor with or without menthol under our exposure conditions did not affect the innate visual or perceptual behaviors.

### Normal Spatial Learning and Memory in Mice With Long-Term Exposure of Electronic Nicotine Delivery Systems With or Without Menthol Flavor

**FIGURE 6:**
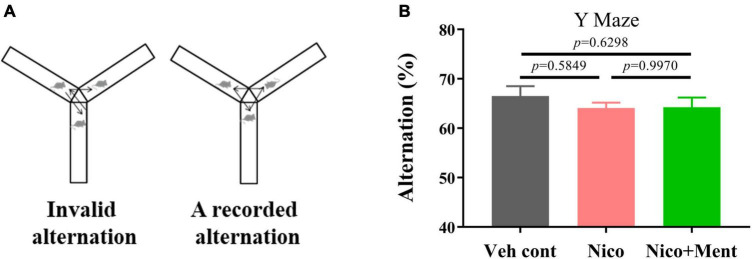
Spatial memory assessment in mice exposed to long-term vapor exposure. **(A)** Schematic diagram of recorded alternation of one trial in Y-maze. Spontaneous alternation was defined as successive entries into the three different arms (without returning to any arm). **(B)** The percentage alternation was calculated as the ratio of actual to possible alternations (the total number of arm entries - 2) × 100. The value of *p < 0.05 as determined by ordinary one-way ANOVA and multiple comparisons with every other group. Bars represent marginal means ± SEM. *N* = 8 per group. Veh cont, Vehicle control; Nico, Nicotine; Nico + ment, nicotine with menthol flavor.

Previous studies have suggested a potential relationship between social activity and the overall executive functioning, working memory, and visuospatial abilities in healthy older adults (Kelly et al., 2017; Kuiper et al., 2017; Perry et al., 2021). Here, we used the Y-maze test to measure the cognition and spatial memory of mice after exposure to e-cigarette vapor. The percentage of alternation in Y-maze arms was analyzed (Figures 6A,B). There were no significant changes of alternation [*F*_(2, 21)_ = 0.6216, *p* = 0.5467] in the mice of either Nico group (64.07 ± 1.119 %) or Nico + ment group (64.25 ± 1.973 %) compared to the PG/VG group (66.53 ± 1.978 %). These data indicated that menthol flavor combined with nicotine in e-cigarette had no effect on spatial learning and memory.

### The Reduced Adenosine 5′ Monophosphate-Activated Protein Kinase Activation in the Hippocampus by Chronic Electronic Nicotine Delivery Systems Exposure With Menthol Flavor

**FIGURE 7:**
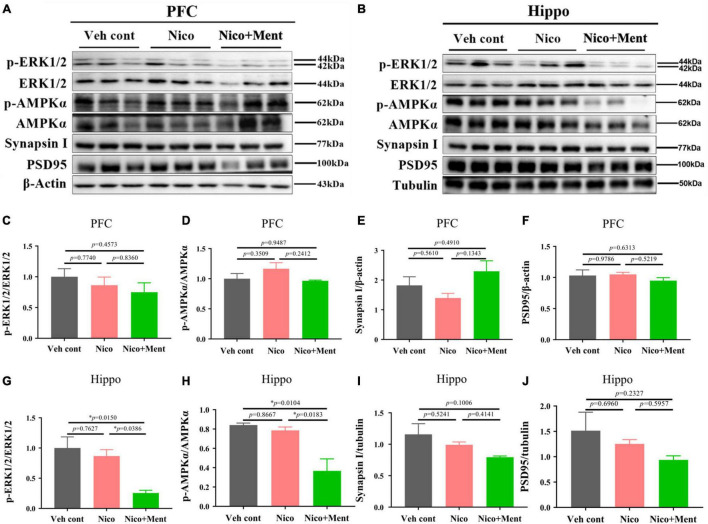
The immunometabolic signals and synaptic protein analyses in the PFC/hippocampus of mice after long-term vapor exposure. Western blot analyses of the expressions of p/t-ERK1/2, p/t-AMPKα, synaptic proteins, such as Synapsin-1 and PSD95 in the PFC **(A)** and the hippocampus **(B)**. The quantification data of the above molecules are presented in **(C–J)**, respectively. Data are expressed as means ± SEM. The values of *p < 0.05 as determined by ordinary one-way ANOVA and multiple comparisons with every other group. Veh cont, Vehicle control; Nico, Nicotine; Nico + ment, nicotine with menthol flavor.

We further investigated the underlying mechanisms of the effects of chronic vapor on nicotine with or without menthol flavor. The prefrontal cortex and the hippocampus are the interconnected brain regions and hubs for modulating high brain functions and neuropsychiatric behaviors especially social behaviors (Padilla-Coreano et al., 2016; Eichenbaum, 2017). We here analyzed the AMPK/ERK signaling pathways which are involved in neuronal metabolism, neuroinflammation, and synaptic plasticity. By using Western blotting, we found that the hippocampal activation of ERK1/2 (as shown by phosphorylated/total ERK1/2) and AMPKα (as presented by phosphorylated/total AMPKα) in the hippocampus was decreased by menthol flavor when added into the nicotine vapor (Figures 7B,G,H). Further, we also evaluated the expression levels of presynaptic protein, synaptin-1, and the postsynaptic protein, PSD95 in the prefrontal cortex and the hippocampus to assess the alterations in synaptic plasticity. A slight reduction of synapsin-1 was observed in the hippocampal region without statistical significance (Figure 7I). The AMPK/ERK signaling in PFC (Figures 7A,C,D), and the expressions of Synapsin I and PSD95 in PFC and the hippocampus were not significantly changed under our vapor exposure condition (Figures 7A,B,E,F,I,J). These data suggested that the menthol flavor in ENDS might inactivate the AMPK-ERK signaling in the hippocampus.

### Alterations of Peripheral Cytokine Levels Responded to Menthol Flavor in Electronic Nicotine Delivery Systems

**FIGURE 8:**
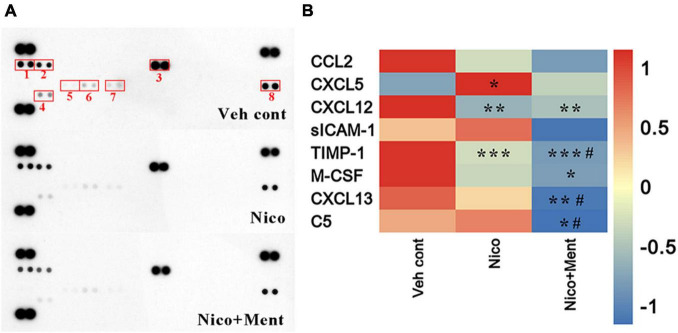
The cytokine profile in the sera is altered by long-term vapor exposure. **(A)** Representative blots from serum samples of mice treated with long-term vapor exposures of vehicle control, ENDS, or ENDS with menthol flavor that presented levels of 40 known cytokines using the murine Proteome Profiler Cytokine Array. Spots showing differential expression are boxed. (1): CXCL13; (2): C5; (3): sICAM-1; (4): TIMP-1; (5): CXCL5; (6): M-CSF; (7): CCL2; (8): CXCL12. **(B)** Heatmap of quantification revealed significant changes greater than 1.2-fold, data were determined by ordinary one-way ANOVA and multiple comparisons with every other group. *p < 0.05, **p < 0.01, ***p < 0.001 compared to the Veh cont; ^#^*p* < 0.05 compared to Nico group, accordingly. Veh cont, Vehicle control; Nico, Nicotine; Nico + ment, nicotine with menthol flavor.

Multiple cytokines and chemokines have been investigated regarding their roles in neuropsychiatric behaviors (Kronfol and Remick, 2000; Polacchini et al., 2018). No significant differences in weight gain among all the treatment groups were observed (Supplementary Figure 2). Here, we profiled multiple cytokines in the sera to assess the peripheral effects of chronic ENDS vapors which might respond to the social behavioral changes. Forty cytokines were measured in our experiment and we observed that the sera levels of CXCL12 and TIMP-1 were significantly reduced while that of the CXCL5 was dramatically increased after nicotine vapor exposure, and a further decline of TIMP-1 and CXCL13 were detected in the group of menthol-flavored ENDS compared to the Nicotine alone group. The serum expressions of CXCL12 were decreased in vapor groups with or without menthol flavor compared to the Veh control group, suggesting that the menthol flavor had no additional effects in ENDS on the serum level of CXCL12. The sera level of M-CSF was only reduced in Nico + ment group compared to the Vehicle control group. The C5 level in the sera was found dramatically decreased in the vapor group of nicotine with menthol flavor compared to either vehicle or ENDS exposure of nicotine only, suggesting a strong downregulation of C5 in the sera of menthol flavorings in ENDS (Figures 8A,B). These data suggested that menthol flavor may modulate the serum expressions of cytokines that responded to the alteration on the social activity by ENDS.

### Linear Correlations Among the Central/Peripheral Immunometabolic Indices and the Response of Social Behaviors to Menthol Flavor in Electronic Nicotine Delivery Systems

**FIGURE 9:**
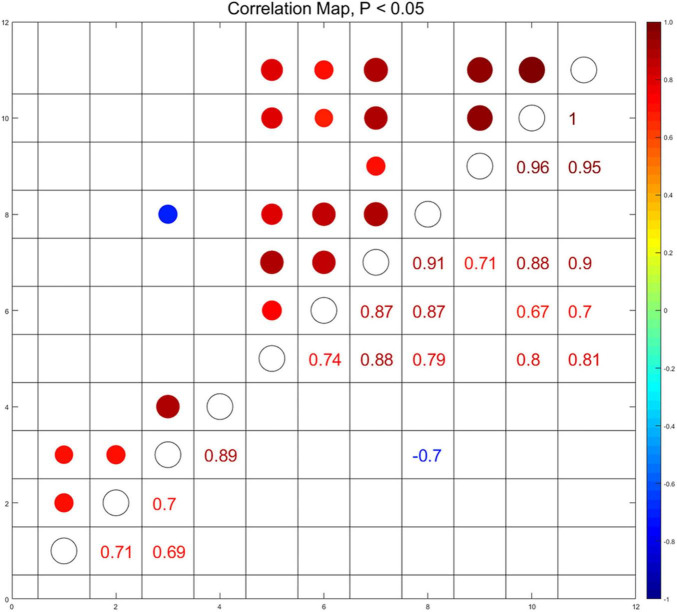
Correlations among immunometabolic signals presented as the activation of AMPK-ERK in the PFC and the hippocampus, cytokines in the sera, and the social behavioral parameters *(p* < 0.05). Through pairwise comparison of the social behavioral index and biochemical indicators, we selected and displayed those with *p*-value less than 0.05, and used the area and color depth of the dot to represent the *r*-value. (1): Time around the target in the sociability session of Three-Chamber Test (TCT); (2): Preference index in the sociability session; (3): Time around the target in the test session of preference for social novelty; (4): Preference index in the test session of preference for social novelty; (5): the level of p-ERK/ERK in the hippocampus; (6): the level of p-AMPKα/AMPKα in the hippocampus; (7–11): the serum level of CXCL13 (7), C5 (8), CXCL12 (9), M-CSF (10), and TIMP-1 (11).

To elucidate the correlation among the social activity and the immunometabolic indices in the hippocampus and in the sera which were affected by menthol flavor in ENDS, we conducted the Spearman’s correlation analysis among social behavioral indices that activated the AMPK in the hippocampus and the cytokine levels in the sera (*p* < 0.05, Figure 9). The serum level of C5 was found to be negatively correlated with the preference for social novelty as measured by the target exploration time in Stage 2 (*r* = -0.7). The serum level of C5 was also positively correlated with the activation of ERK (p-ERK/ERK, *r* = 0.79) and AMPK (p-AMPK/AMPK, *r* = 0.87) in the hippocampus. The levels of M-CSF and TIMP-1 in the sera were also found to be correlated with the AMPK-ERK signaling in the hippocampus (Figure 9). This set of correlation data suggested that the menthol flavor in ENDS may induce comprehensive immunometabolic responses in the brain nuclei and in the sera corresponding to the social activity change.

## Discussion

The e-cigarette is among the focus of controversy since it may be harm-reducing for traditional smokers seeking to quit, while harm-initiating for former or never smokers, particularly among the youth (Kalkhoran and Glantz, 2016; Fairchild et al., 2018; Prochaska, 2019). The US FDA has been seeking to reduce nicotine concentrations in conventional tobacco cigarettes to non-addictive levels while emphasizing other nicotine delivery products, such as the role of e-cigarettes in attenuating the harmful effects of combustible tobacco (McCarthy, 2017). However, further scientific evidence is needed to convince the safety of e-cigarette and their aid on smoking cessation, given the widespread use of chemicals/artificial flavors to mimic natural flavors commonly used in the e-cigarettes.

A wide variety of flavor options of e-cigarettes on the market has grown in popularity and entices young generations to smoke (Zare et al., 2018). The menthol flavor is among the most commonly used flavorings in e-cigarettes, and an exception in which the flavored e-cigarettes have been banned by Federal regulations recently (Kaur et al., 2020). It has been well-documented that e-cigarettes cause systemic toxicity, including lung and liver injuries; the cytotoxicity induced by e-cigarette flavoring chemicals has also been determined in cell lines and humans. Repeated exposure to menthol was found to significantly decrease cell viability (Rickard et al., 2021); therefore additional research is urged to understand the mechanisms of the toxicity of flavorings and the chemical combinations in ENDS.

Smoking may increase the risk of mental disorders and non-affective psychoses. A systematic review of literature from 1946 to 2017 followed by a meta-analysis suggested that chronic tobacco smoking was strongly associated with neuropsychological deficits and cognitive impulsivity (Conti et al., 2019). The e-cigarette products in the market are generally composed of nicotine with flavor. Menthol flavor was the top choice among teen vapers according to research in the US (Leventhal et al., 2019). In this case, it is necessary to understand the neuropsychiatric roles and their effects on the brain as well as the peripheral system of menthol-flavored e-cigarettes. However, there is limited evidence on the neuropsychiatric roles of menthol flavor in ENDS that was specifically evaluated *in vivo* in animal models or humans.

Social behaviors are fundamental for the survival of any vertebrate species. Epidemiological data indicated that smokers endorse socializing as a reason to smoke (Fidler and West, 2009) and social functioning was found enhanced in smokers which was supposed to be related to nicotine (Martin and Sayette, 2018). However, very limited work has been implemented to depict the effect of e-cigarettes on social functions in animal models to reveal the underlying molecular mechanisms. In our current work, we have used a standard and precise vapor device to mimic the exposure of ENDS, which avoided the restraint stress that might have been caused by the nose-only aerosol exposure, and evaluated the social behaviors after long-term exposure with daily 30-min inhalation. As shown in the previous study, the effects of nicotine on social behaviors are complex regarding the dose, the schedule of administration, housing, and individual differences; nicotine may increase the social interaction at low doses but reduce it at high doses (Blanco-Gandia et al., 2015); and it was also presented to improve the sociability and reduced repetitive behaviors in a mouse model of autism at certain doses while no effects were observed in the normal mice (Mahmood et al., 2020). Consistent with some of these literature, we found in our experiment that, although the slight decrease in the social activity (no statistical difference) was observed in nicotine-vapored mice, the long exposure to menthol flavored ENDS was found to have compensatory enhancing effects on the sociability and preference for social novelty compared to the vapor exposure of nicotine alone, suggesting the antagonistic effect on the social functioning of menthol flavoring as a combinational ingredient with nicotine in ENDS.

Since social behaviors are instinctive with flexibility (Wei et al., 2021), and influenced by other psychiatric behaviors, we further assessed the behaviors related to emotion, cognition, and innate state in the mice after ENDS exposure with or without menthol flavor. Interestingly, under our ENDS exposure condition, which was a short-term treatment per day and it lasted for the long term; the ENDS exposure with menthol flavor did not change the anxiety/depressive-like behaviors measured by the elevated plus maze and tail suspension test; the innate visual or perceptual behavioral responses measured by the VLT, HPT, and the spatial memory evaluated by Y-maze in mice. One limitation of this study was that only male mice were used with a limited sample size. It will be necessary to understand how female individuals cope with e-cigarettes and to further confirm the complex interplay of menthol flavoring with nicotine in ENDS.

Further, we characterized the central and peripheral changes induced by the vapors of nicotine alone or combined with menthol flavor that may be related to the alterations in social activities. The prefrontal cortex and hippocampus are hub regions that are dominant in many complex behaviors including social activities and social cognition (Preston and Eichenbaum, 2013; Li et al., 2020; Qi et al., 2021). Increasing attention has been paid to the role of the crosstalk between metabolic, inflammatory, and neuropsychiatric disorders (Culmsee et al., 2018), such as the activation of ERK and the AMPK levels (Camargo et al., 2021). The adenosine 5′ monophosphate-activated protein kinase (AMPK) is a heterotrimeric serine/threonine kinase that promotes ATP generation and is regarded as a key regulator of cellular energy metabolism and mitochondrial homeostasis (Chaube and Bhat, 2016; Herzig and Shaw, 2018). Therefore, we measured the immunometabolic molecular signals in the prefrontal cortex, hippocampus, and the cytokines in the sera altered by nicotine vapor with or without menthol flavor and investigated their correlations with social behavioral changes. Our results suggested a negative correlation of preference for social novelty and C5 level in the sera of mice. Further, the serum level of C5 was also found to positively correlate with the activation of AMPK-ERK signaling in the hippocampus, which may hint at a coordinated response in the central and peripheral system to ENDS which contribute to the social behavioral enhancement induced by the menthol flavor. Previous studies have shown that anxiety was associated with low levels of many cytokines in sera, such as CCL11, CCL2, CCL5, and IL-6; and lower peripheral levels of CXCL5 was observed in people with psychiatric disorders, such as schizophrenia and recurrent depressive disorder with suicidal ideation (Polacchini et al., 2018). The cytokine profile observed in our study indicated that the menthol flavor in ENDS may act to reverse the potentially reducing effects of nicotine on social activities.

In conclusion, our present study profiled the social behaviors modulated by menthol flavor in ENDS. We presented the compensatory enhanced social activity induced by menthol flavor in the nicotine-containing e-cigarette. The striking enhancement in social activity induced by menthol flavoring, in combination with nicotine in ENDS, may explain the increased severity of nicotine dependence in menthol-flavored e-cigarette vaporer and the popularity of menthol/mint-flavored e-cigarettes in the market. The ENDS induced the immunometabolic alternations in the hippocampus, as well as in the sera that correspond to social behavioral changes, suggesting the disruption of systemic homeostasis are only induced by nicotine but also by other flavorings in the e-cigarette. Although our current data indicated the mild influences of the neuropsychiatric behaviors in the mice due to long-term ENDS exposure with daily intake limit; the phenomenon of enhanced social functions induced by menthol flavor in ENDS highly alerts us with the information that e-cigarette flavoring additives may have complex interplay with nicotine and lead to increased addiction as well as immunometabolic disruption among the e-cigarette users who definitely need further investigations.

## Data Availability Statement

The raw data supporting the conclusions of this article will be made available by the authors, without undue reservation.

## Ethics Statement

All animal experiments and procedures were carried out in accordance with protocols approved by the Animal Care and Use Ethics Committee of the Shenzhen Institutes of Advanced Technology, Chinese Academy of Sciences.

## Author Contributions

X-AL designed the experiments. ZX, ZM, A-XL, JT, JL, and JW performed the experiments. ZX, YT, CD, ZC, A-XL, X-YJ, JT, and XL performed the data analyses. X-AL, ZC, YT, A-XL, JT, and XG contributed to the manuscript writing. TL, ZC, ZL, LW, and SL revised the manuscript. All authors have read and approved the manuscript.

## Conflict of Interest

The authors declare that the research was conducted in the absence of any commercial or financial relationships that could be construed as a potential conflict of interest.

## Publisher’s Note

All claims expressed in this article are solely those of the authors and do not necessarily represent those of their affiliated organizations, or those of the publisher, the editors and the reviewers. Any product that may be evaluated in this article, or claim that may be made by its manufacturer, is not guaranteed or endorsed by the publisher.

## Supplementary Material

Supplementary Figure 1 Urine cotinine measured during vapor exposure. The urine samples from the mice of all exposure groups were collected immediately following the daily exposure period and assessed by Liquid chromatography-mass spectrometry (LC-MS/MS) for cotinine as a marker for the intake of nicotine-containing vapors. Data are expressed as group mean ± standard error. **p* < 0.05, ^**^*p* < 0.001 as determined by ordinary one-way ANOVA and multiple comparisons with every other group. Veh cont, Vehicle control; Nico, Nicotine; Nico + ment, nicotine with menthol flavor.

Supplementary Figure 2 Weight measured weekly during vapor exposure. **(A)** Weight measured weekly from week 0 to week 12. **(B)** Delta weight gain corrected for physiological growth, as measured in the weight value of the following week minus the weight value of the first week. Data are shown as mean ± SEM. **p* < 0.05. Veh cont, Vehicle control; Nico, Nicotine; Nico + ment, nicotine with menthol flavor.

Supplementary Table 1 Device parameters of e-cigarette vapor exposure program.

Supplementary Table 2 Primary antibodies used for Western blot in this study.
