# Corrigendum: Menthol Flavor in E-Cigarette Vapor Modulates Social Behavior Correlated With Central and Peripheral Changes of Immunometabolic Signalings

**DOI:** 10.3389/fnmol.2022.913285

**Published:** 2022-05-12

**Authors:** Zhibin Xu, Ye Tian, A.-Xiang Li, Jiahang Tang, Xiao-Yuan Jing, Chunshan Deng, Zhizhun Mo, Jiaxuan Wang, Juan Lai, Xuemei Liu, Xuantong Guo, Tao Li, Shupeng Li, Liping Wang, Zhonghua Lu, Zuxin Chen, Xin-an Liu

**Affiliations:** ^1^Guangdong Provincial Key Laboratory of Brain Connectome and Behavior, CAS Key Laboratory of Brain Connectome and Manipulation, Brain Cognition and Brain Disease Institute (BCBDI), Shenzhen Institute of Advanced Technology, Chinese Academy of Sciences, Shenzhen, China; ^2^Shenzhen-Hong Kong Institute of Brain Science-Shenzhen Fundamental Research Institutions, Shenzhen, China; ^3^University of Chinese Academy of Sciences, Beijing, China; ^4^Department of Forensic Medicine, School of Medicine, Xi'an Jiaotong University, Xi'an, China; ^5^State Key Laboratory of Oncogenomics, School of Chemical Biology and Biotechnology, Peking University Shenzhen Graduate School, Shenzhen, China; ^6^Department of Psychiatry, University of Toronto, Toronto, ON, Canada; ^7^Key Laboratory of Modern Toxicology of Shenzhen, Shenzhen Center for Disease Control and Prevention, Shenzhen, China; ^8^Shenzhen Key Laboratory of Drug Addiction, Shenzhen Neher Neural Plasticity Laboratory, The Brain Cognition and Brain Disease Institute, Shenzhen Institute of Advanced Technology, Chinese Academy of Sciences, Shenzhen, China

**Keywords:** e-cigarette, nicotine, menthol, social activity, electronic nicotine delivery systems (ENDS)

In the published article, there was an error in affiliation 1. Instead of “Guangdong Provincial Key Laboratory of Brain Connectome and Behavior, CAS Key Laboratory of Brain Connectome and Manipulation, Brain Cognition and Brain Disease Institute (BCBDI), Shenzhen Institute of Advanced Technology, Shenzhen-Hong Kong Institute of Brain Science-Shenzhen Fundamental Research Institutions, Chinese Academy of Sciences, Shenzhen, China,” it should be “Guangdong Provincial Key Laboratory of Brain Connectome and Behavior, CAS Key Laboratory of Brain Connectome and Manipulation, Brain Cognition and Brain Disease Institute (BCBDI), Shenzhen Institute of Advanced Technology, Chinese Academy of Sciences, Shenzhen, China.”

In the published article, there was an error regarding the affiliation(s) for Zhibin Xu. As well as having affiliation(s) 1, they should also have “Shenzhen-Hong Kong Institute of Brain Science-Shenzhen Fundamental Research Institutions, Shenzhen, China.”

In the published article, there was an error regarding the affiliation(s) for Zhonghua Lu, Ye Tian and Liping Wang. As well as having affiliation(s) 1, they should all also have “University of Chinese Academy of Sciences, Beijing, China.”

In the published article, there was an error regarding the affiliation(s) for Xin-an Liu. As well as having affiliation(s) 2, they should also have “Guangdong Provincial Key Laboratory of Brain Connectome and Behavior, CAS Key Laboratory of BrainConnectome and Manipulation, Brain Cognition and Brain Disease Institute (BCBDI), Shenzhen Institute of Advanced Technology, Chinese Academy of Sciences, Shenzhen, China,” and also “Shenzhen-Hong Kong Institute of Brain Science-Shenzhen Fundamental Research Institutions, Shenzhen, China.”

The authors apologize for this error and state that this does not change the scientific conclusions of the article in any way. The original article has been updated.

## Publisher's Note

All claims expressed in this article are solely those of the authors and do not necessarily represent those of their affiliated organizations, or those of the publisher, the editors and the reviewers. Any product that may be evaluated in this article, or claim that may be made by its manufacturer, is not guaranteed or endorsed by the publisher.

