# Supplementary figures and images for: Menthol Flavor in E-Cigarette Vapor Modulates Social Behavior Correlated With Central and Peripheral Changes of Immunometabolic Signalings
Source: Front Mol Neurosci. 2022 Mar 10;15:800406. doi: 10.3389/fnmol.2022.800406 (PMC8960730; doi:10.3389/fnmol.2022.800406)

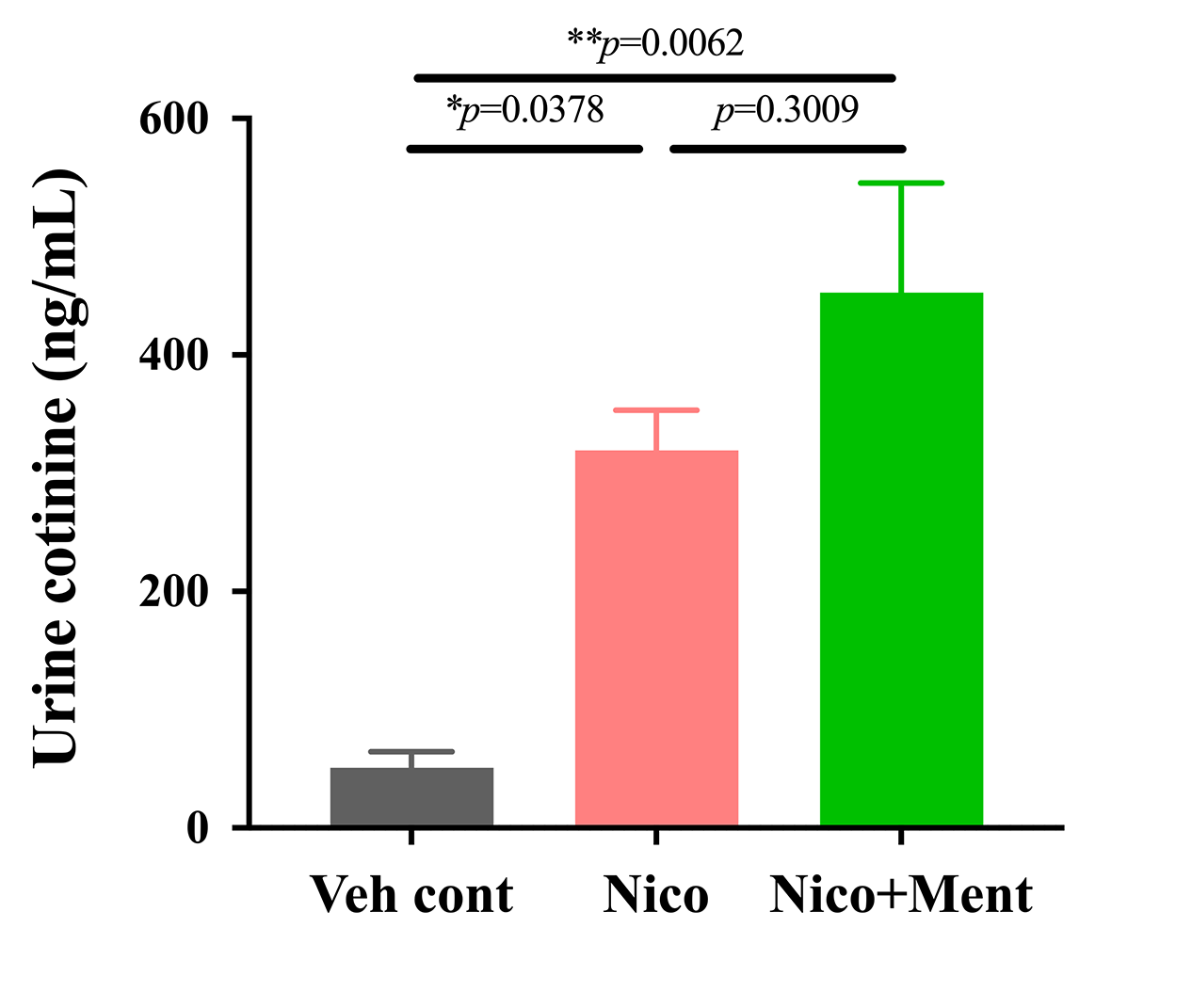

Supplement: Supplementary Figure 1 — Urine cotinine measured during vapor exposure. The urine samples from the mice of all exposure groups were collected immediately following the daily exposure period and assessed by Liquid chromatography-mass spectrometry (LC-MS/MS) for cotinine as a marker for the intake of nicotine-containing vapors. Data are expressed as group mean ± standard error. *p < 0.05, **p < 0.001 as determined by ordinary one-way ANOVA and multiple comparisons with every other group. Veh cont, Vehicle control; Nico, Nicotine; Nico + ment, nicotine with menthol flavor. [file Image_1.TIFF]

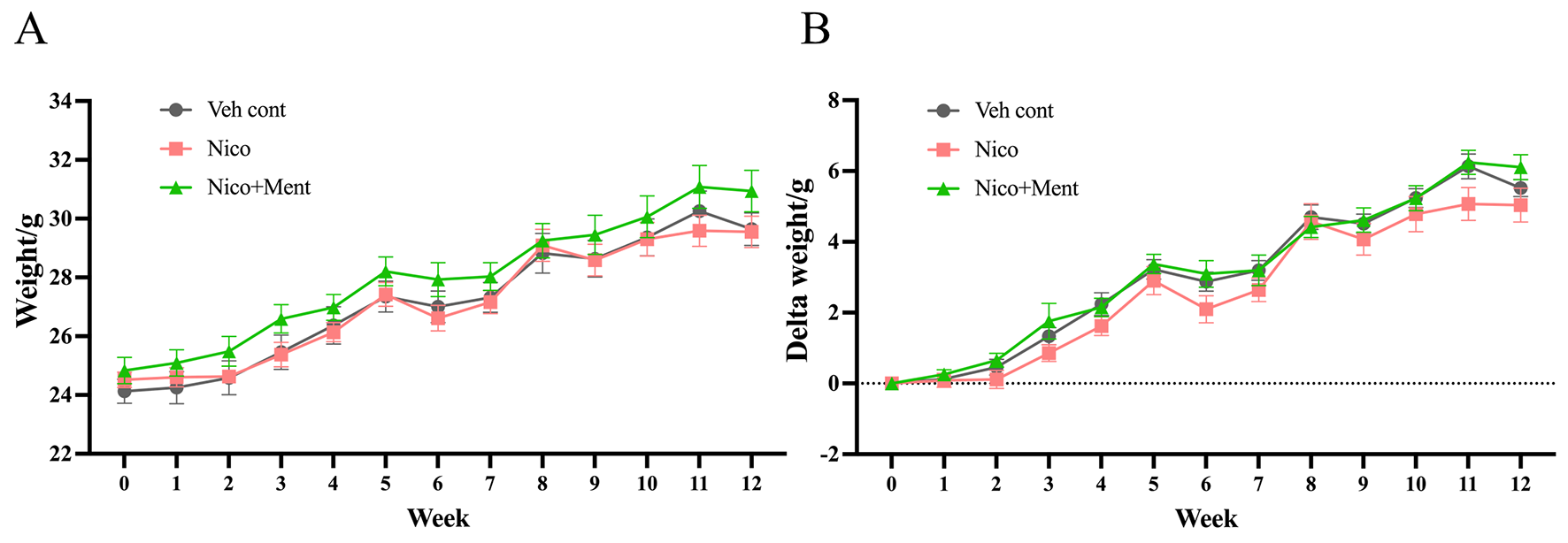

Supplement: Supplementary Figure 2 — Weight measured weekly during vapor exposure. (A) Weight measured weekly from week 0 to week 12. (B) Delta weight gain corrected for physiological growth, as measured in the weight value of the following week minus the weight value of the first week. Data are shown as mean ± SEM. *p < 0.05. Veh cont, Vehicle control; Nico, Nicotine; Nico + ment, nicotine with menthol flavor. [file Image_2.TIFF]
