# Supplementary material for: Menthol Flavor in E-Cigarette Vapor Modulates Social Behavior Correlated With Central and Peripheral Changes of Immunometabolic Signalings
Source: Front Mol Neurosci. 2022 Mar 10;15:800406. doi: 10.3389/fnmol.2022.800406 (PMC8960730; doi:10.3389/fnmol.2022.800406)
Supplement: Supplementary Table 1 — Device parameters of e-cigarette vapor exposure program. [file Table_1.pdf]

Supplemental Tables

Table S1. Device parameters of e-cigarette vapor exposure program.

FlexiWare program	IX-2PD-4DIO-ECIG inExpose			
Program running time	30 min per time / day			
Gas Flow Rate	2 L/min			
	Simusoid	Clear	Constant I	Constant II
Procedural priming	0.5	1.5	4	0
Program breakpoint	3.5	2.5	30	30
Amplitude	1.81	1.57	2 L/min	2 L/min

Table S2. Primary antibodies used for Western blot in this study.

Antibody	Vendor	Cat no.	Working dilution
p-ERK1/2	CST	#4370	1:1000
ERK1/2	proteintech	16443-1-AP	1:1000
p-AMPKα	CST	#2535	1:1000
AMPKα	CST	#5832	1:1000
Synapsin 1	abcam	ab254349	1:1000
PSD95	abcam	Ab18258	1:1000
β-Actin	Santa Cruz	sc-47778	1:500
tubulin	Santa Cruz	sc-8035	1:500
